# Investigating the Efficacy of the Hand Selection Complexity Task Across the Lifespan

**DOI:** 10.3389/fpsyg.2019.01130

**Published:** 2019-06-11

**Authors:** Nicole Williams, Sara M. Scharoun Benson, Pamela J. Bryden

**Affiliations:** ^1^ Department of Kinesiology and Physical Education, Wilfrid Laurier University, Waterloo, ON, Canada; ^2^ Department of Kinesiology, University of Windsor, Windsor, ON, Canada

**Keywords:** handedness, hand preference, hand selection, task complexity, lifespan

## Abstract

There is inconsistent evidence demonstrating a relationship between task complexity and hand preference. However, analyzing the point at which task complexity overrides the decision to demonstrate a biomechanically efficient movement can enable complexity to be quantified. Young children (ages 3–7), adolescents (ages 8–12), young adults (ages 18–25), and older adults (ages 65+) performed a newly developed Hand Selection Complexity Task (HSCT) and completed the Waterloo Handedness Questionnaire (WHQ). The HSCT included a reciprocal Fitts’ tapping task performed in the contralateral space (i.e., same side as preferred hand), followed by ipsilateral space (i.e., opposite side of preferred hand). An alternating contralateral-ipsilateral pattern enabled the participant to progress through six levels of difficulty in three conditions (manipulating target amplitude, width, and combined factors). As participants were free to perform with whichever hand (i.e., preferred, non-preferred) they deemed most appropriate, the level of difficulty where a hand switch occurred was identified. HSCT completion time and error scores were also computed. Findings revealed age to be a significant predictor of dependent measures when considering significant effects and interactions. Combined with the covariate WHQ score as a significant predictor of HSCT time and errors (in some, but not all cases), it can be argued that age-related effects reflect the development of handedness, and changes in strength of handedness across the lifespan. Together, findings suggest that task complexity plays an important role in hand selection when performing a task of increasing difficulty. It appears that task complexity will take precedent over object proximity and biomechanical efficiency, at a certain point, in order to complete the movement with the preferred hand. This point ultimately changes throughout the lifespan.

## Introduction

Handedness is the hand that individuals not only prefer to use, but also the one that performs unimanual tasks more efficiently (Steenhuis and Bryden, 1999; Corey et al., 2001). Approximately, 90% of the population prefer their right hand; however, the size of the preferred hand advantage is influenced by various task characteristics (Steenhuis and Bryden, 1989; Hausmann et al., 2004). The preferred hand is chosen more often, and the preferred-hand advantage is greater for more difficult (i.e., more highly skilled or complex) tasks. When task complexity is high (Hausmann et al., 2004), the preferred-hand system is more efficient and specialized for utilizing visual feedback (e.g., Flowers, 1975) and for controlling specific aspects of motor output such as limb dynamics (Sainburg, 2002). Unfortunately, researchers tend to utilize different definitions of “task complexity” based on task characteristics (e.g., precision requirements, number of action steps, etc.). As there is no explicit definition of task complexity, it is very difficult to quantify.

One of the first documented studies on hand performance and task complexity compared right and left-handers on a simple (rhythmical tapping) and a complex (manual aiming) task, performed with both hands (Flowers, 1975). Given that participants were unable to monitor visually or to make visual corrections during the simple task, it was argued to be ballistic, while the manual aiming task was closed-loop as it required participants to make visual corrections. The results indicated trivial differences in performance between the hands for the simple task. However, for the complex task, significant differences between the hands were found for both movement time and accuracy measurements (Flowers, 1975).

A few years later, the performance of the two hands was compared using a pegboard (Annett et al., 1979). Participants moved a row of 10 wooden dowels from one row of holes to another row closer to the participant. Hole-to-peg ratio was manipulated using Fitts Law (Fitts, 1954). As the ratio decreased (i.e., task difficulty increased), the temporal difference between the hands became significantly larger. The difference between the hands was a result of the non-preferred hand making significantly more errors—contacting the surface around the hole before inserting the peg—than the preferred hand. Unexpectantly, no significant differences were found between the hands for the time to insert a peg or the overall movement speed. It was argued the difference between the hands was due to the variability of the non-preferred hand when performing the task (Annett et al., 1979).

These early studies (Flowers, 1975; Annett et al., 1979) suggested performance differences between the hands vary as a function of task difficulty. Other, more recent work continues to show small but significant differences between the hands for performance tasks (e.g., McManus et al., 2016). Related research has also examined how task complexity influences hand selection. More specifically, a preferential reaching task was developed to allow task difficulty to be manipulated, while the proximity of the reaches involved in the task were maintained (Bryden et al., 2000). In the original work (Bryden et al., 2000), the goal of the task (e.g., pointing, picking up, knocking over, sweeping, tossing, and placing the object) was manipulated. No differences in hand selection as a function of task complexity were found, although it was found that both right and left-handed individuals were more likely to use their preferred hand during reaches in ipsilateral space and at the midline. A similar lack of an effect of task complexity was found in a cross-sectional study examining developmental changes in hand selection (Bryden and Roy, 2005). However, when the preferential reaching task was altered to use complex tools, rather than dowels or toys, significant effects of tasks complexity on hand selection were noted (Mamolo et al., 2005, 2006). Such findings raise the question of how to best define and quantify task complexity.

More recently (Gooderham and Bryden, 2014), the preferential reaching task was modified to include gradients comprised of eight tasks of increasing complexity. Notably, however, task difficulty was not manipulated in a quantifiable manner, but rather based on the authors perceptions. Gradients were positioned in ipsilateral and contralateral space, with the participant seated at the midline. Starting with the contralateral gradient, participants were instructed to perform the task with the hand that felt most comfortable. Upon completion, the ipsilateral gradient was performed, with the same instructions. The remaining seven tasks progressed in this manner. The “highest” task performed before participants switched to use the opposite hand for both contralateral and ipsilateral space was recorded (Gooderham and Bryden, 2014).

Results indicated that all age groups (young children ages 2–4, adolescents ages 10–14, young adults, and older adults over age 65) performed with the preferred right hand in ipsilateral space. In contralateral space, children performed the highest number of tasks (6 out of 8) with the non-preferred hand compared to adolescents who completed the fewest tasks (1.6 out of 8) with the non-preferred hand. In comparison, young adults and older adults performed similarly in contralateral space, completing approximately half of the gradients with the non-preferred hand. Findings were concurrent with previous cross-sectional studies investigating developmental effects in children (e.g., Bryden et al., 2000, 2011; Bryden and Roy, 2005; Carlier et al., 2006; Scharoun and Bryden, 2014), where young children typically use the hand that corresponds to the side of space and older children adopt an inflexible pattern of responding with their preferred hand. Literature assessing older adults is inconsistent, such that researchers have reported an increase (Weller and Latimer-Sayer, 1985), a decrease (Kalisch et al., 2006), and no change whatsoever in the strength of hand preference (Schaefer, 2015). Similar to Gooderham and Bryden (2014), Schaefer (2015) noted similar performance to young adults by the older adults.

Gooderham and Bryden (2014) were also successful in identifying the point where individuals switch their hand selection from preferred to non-preferred for tasks of increasing difficulty. However, the major limitation was that task difficulty was not quantified and did not necessarily progress in a linear stepwise function. To better understand the role of task complexity in hand selection, the current study implemented a paradigm similar to Gooderham and Bryden (2014) with young children (ages 4–7), adolescents (ages 8–12), young adults (ages 18–25), and older adults (ages 65+).

In the current study, Fitts Law (Fitts, 1954) was used to manipulate task difficulty in a quantifiable manner. Three conditions, which altered target width, amplitude, and a combination of width and amplitude, were implemented to discern how each individual factor, and both factors combined, impact performance. This was done in consideration of the notion that, although amplitude changes affect movement time, they do not affect precision. That said, manipulating target width does affect precision, which is more likely to influence hand selection. It was hypothesized that the combined condition would reveal the most notable effects. The objective was to identify the point at which people shift their hand selection from the non-preferred to the preferred hand in contralateral space. This “switch” would reflect the point at which task complexity overrides biomechanical efficiency. It was hypothesized that tasks in contralateral space would be performed with the non-preferred hand and tasks in ipsilateral space with the preferred hand (Gabbard et al., 1997, 2003), based on object proximity. However, as the level of difficulty increased (i.e., increased task complexity), participants would opt for less biomechanically efficient movements (Bradshaw et al., 1994), maintaining preferred hand to ensure successful task completion. Finally, it was hypothesized that age would be a significant covariate.

## Materials and Methods

### Participants

As described in Williams (2014), this study included 80 participants (*M* = 31, *F* = 49), in four age groups (Table 1). Young, typically developing children (ages 4–7) and adolescents (ages 8–12), were from a local child development center, and a summer camp at Wilfrid Laurier University. Young adults (ages 18–25) were undergraduate and graduate students at Wilfrid Laurier University. Older adults, all of whom were over the age of 65, were recruited from a local retirement home. Participants with motor deficits that may have impacted dexterity and cognitive deficits that may have affected comprehension were excluded. Participants with mild- to -moderate arthritis were not excluded; however, those with severe arthritis were. All adult participants and parents/guardians of participating children provided voluntary, written informed consent. The Research Ethics Board at Wilfrid Laurier University reviewed and provided full ethical clearance for the research. It should also be noted that the current experiment was included as a portion of a thesis completed by Williams (2014).

**Table 1:** Participant demographics according to group: Age (Mean: Standard Deviation), Male: Female, Self-report Right handers: Left handers, Total Waterloo Handedness Questionnaire Score.

Group	Age *M* (SD)	*M*:*F*	Self-report RH:LH	WHQ *M*(SD)
Young children	5.46 (1.01)	12:5	12:5	5.36 (11.98)
Adolescents	10.03 (0.91)	10:21	28:3	20.10 (17.23)
Young adults	22.65 (1.42)	8:12	20:0	28.60 (5.12)
Older adults	77.17 (5.59)	1:11	11:1	26.42 (17.82)

### Apparatus and Procedures

**Figure 1:**
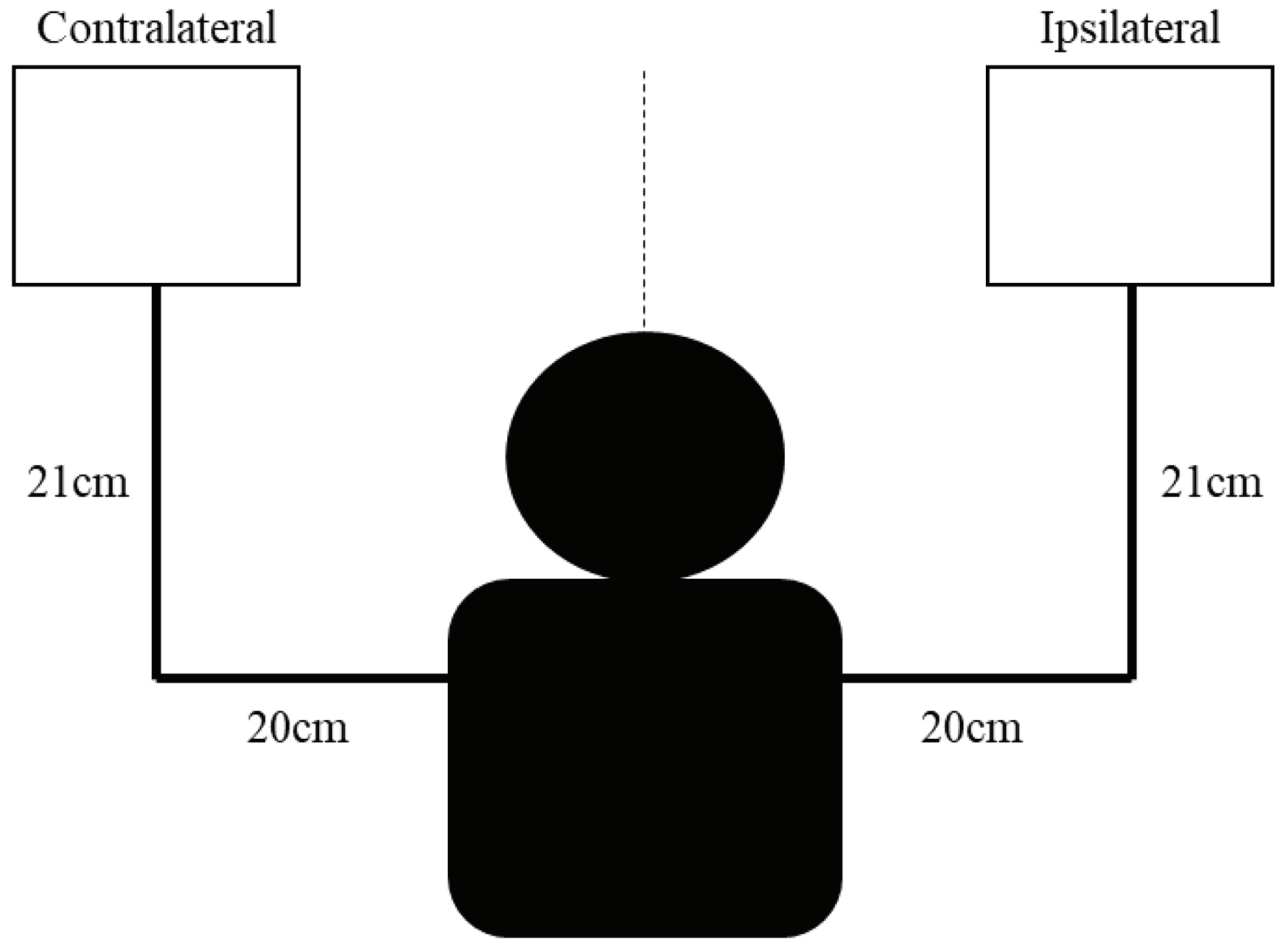
Schematic representation of the Hand Selection Complexity Task. Here, a right-handed participant is seated at the midline with hands resting comfortably in front of them. An identical task gradient is positioned in contralateral and ipsilateral space, 20 cm laterally and 21 cm anteriorly.

Participants performed the Hand Selection Complexity Task (Figure 1) and, subsequently, the Waterloo Handedness Questionnaire to prevent any undue bias (i.e., consideration of hand preference) that the opposite order may have caused.

#### Hand Selection Complexity Task

Created as an observational assessment (Figure 1), participants were seated at a table for the duration of the task. Each trial commenced with both hands resting comfortably on the table at the midline. Identical gradients, displayed on white letter paper (21.6 cm × 27.9 cm) in black ink, were placed in ipsilateral (right space for right handers and left space for left handers) and contralateral space (left space for right handers and right space for left handers), 20 cm from the midline and 21 cm anterior. Six levels of difficulty, determined using Fitts Law (Fitts, 1954), were randomly presented in one of three conditions: (1) Target amplitude: Target width was maintained (1.0 cm), while amplitude (measured from furthest outside point) varied (0.8, 1.2, 2.0, 3.7, 7.2, and 13.8 cm); (2) Target width: Amplitude was maintained (12.8 cm), while target widths varied (8.6, 4.2, 2.2, 1.2, 0.6, and 0.3 cm); and (3) Combined: Both target amplitude (7.3, 10.2, 12.1, 5.1, 3.5, and 6.4 cm), and target width (0.1, 0.5, 1.1, 0.8, 1.8, and 2.7 cm) varied concurrently. Each condition was blocked and counterbalanced. Participants completed a total of 36 trials (6 levels of difficulty × 2 sides × 3 conditions).

Starting with their hand at the midline, participants completed 10 reciprocal tapping movements as quickly and as accurately as possible. Time to completion was recorded from the start signal to the last tap using a stopwatch. The contralateral gradient was performed first, followed by the identical ipsilateral gradient. Participants were free to perform with whichever hand was deemed most appropriate. This alternating pattern (i.e., contralateral than ipsilateral) continued as participants progressed through six levels of difficulty for the three separate conditions. After each gradient was performed, the first author recorded the hand used for task performance. The level of difficulty where a hand switch occurred was recorded as a switch point. For example, if a participant used the non-preferred hand in contralateral space for the first three levels of difficulty and subsequently used the preferred hand for levels fourth through six, the “switch” was recorded at the fourth level. The same was true if participants switched to using their non-preferred hand in ipsilateral space (Gooderham and Bryden, 2014). Errors, which were identified as taps that missed the target, were also recorded (i.e., counted by the researcher and summed upon completion).

#### Waterloo Handedness Questionnaire

Participants were asked to identify hand preference for 20 items using the following scale: left always, left usually, equal, right usually, or right always. A total handedness score was calculated by summing numerical scores, which range from −2 (left always) to +2 (right always) (Table 1; Bryden et al., 2007).

Adult participants completed the questionnaire independently; whereas the questionnaire was read aloud to the young children and adolescents (below the age of 12 years) Explanations of the items were provided, when necessary. Children were asked to identify direction of preference (i.e., right, left, or both) for each item, and how often (e.g. usually or always). This process ensured the same scoring system for all participants. It is important to note that parents of children younger than age 4 were asked to complete the questionnaire on their child’s behalf. Nevertheless, as a result of low response rates (i.e., three missing WHQs), all children were read the questionnaire aloud.

The majority of participants in this study were right-handed. Of the nine participants who self-reported a left-hand preference, eight were children (and one was an older adult). Strength of preference is malleable in childhood (Scharoun and Bryden, 2014), and changes in handedness with aging are not fully understood (Weller and Latimer-Sayer, 1985; Kalisch et al., 2006; Gooderham and Bryden, 2014; Schaefer, 2015). Four young children self-reported a left-hand preference; however, only two (6-year-old male and 7-year-old female) were identified as left handed according to WHQ data. One young child (6-year-old male) who self-reported a right-hand preference, was identified as a very weak left hander. As such, we opted to include all participants.

### Data Analysis

Analyses were conducted using SPSS© version 25.0. Generalized Estimating Equations (GEEs; Liang and Zeger, 1986) were used to examine HSCT completion time, the number of errors, and switch point (i.e., level where a hand switch occurred) data. In the first two models (i.e., HSCT time, number of errors), the effect of condition (amplitude, width, combined), location of the gradient in space (ipsilateral, contralateral), and level of difficulty (ID1–6) were examined. Age and WHQ scores were entered as continuous covariates. Interactions between moderators and covariates examined whether these variables affected the outcome or the slope of change in the outcome. In the third model (switch point), the main effect of condition and level of difficulty were examined. Age and WHQ scores were entered as continuous covariates, and all interactions were examined. The Bonferroni adjustment (*p* < 0.05) was used for multiple comparisons.

## Results

**Figure 2:**
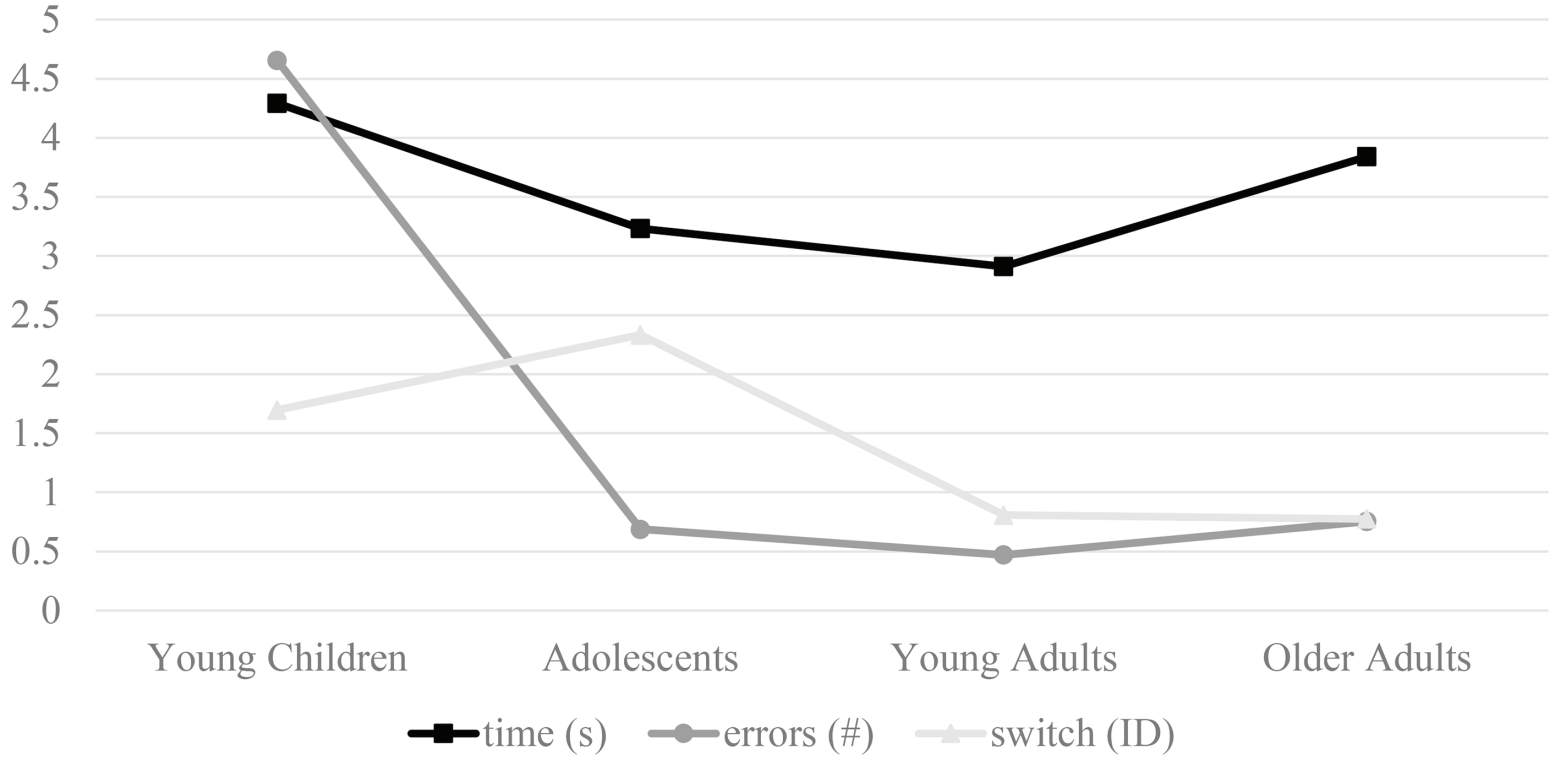
Dependent measures (time, errors, switch point) plotted as a function of age-group.

WHQ score was included as a covariate; therefore, three participants with missing WHQ data were not included in data analysis. To keep the results section concise, the abbreviation “ID” is used for level of difficulty (ID). Furthermore, each level of difficulty is represented by the respective number (ID1–ID6). As the continuous covariate age was a main area of interest, dependent measures are plotted as a function of age (Figure 2) to provide context for the discussion.

### Hand Selection Complexity Task Time

**Figure 3:**
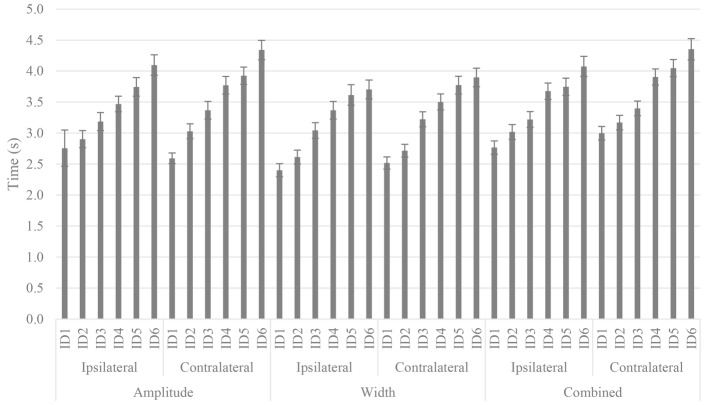
HSCT completion time increased in each task, in each region of space, and in each condition. The covariates age, WHQ score, and the interaction between age and WHQ score were significant predictors of HSCT completion time. Standard error bars are displayed.

A significant three-way interaction between condition, space, and difficulty was found (Wald *χ*^2^(10) = 22.89, *p* = 0.001), with the covariates age (Wald *χ*^2^(10) = 81.21, *p* < 0.001) WHQ score (Wald *χ*^2^(10) = 19.33, *p* = 0.036), and the interaction between age and WHQ score (Wald *χ*^2^(10) = 73.10, *p* < 0.001) as significant predictors. The interaction is displayed in Figure 3; however, for the sake of clarity, only significant main effects and two-way interactions are described in text.

There was a significant effect of space (Wald *χ*^2^(1) = 11.86, *p* = 0.001), with the covariate age (Wald *χ*^2^(1) = 41.14, *p* < 0.001) and the interaction between covariates age and WHQ score (Wald *χ*^2^(1) = 30.96, *p* < 0.001) significantly influencing the effect. Participants were slower in contralateral space (*M* = 3.55, SE = 0.36) compared to ipsilateral space (*M* = 3.39, SE = 0.04; *p* < 0.001). The effect of difficulty was also significant (Wald *χ*^2^(5) = 77.69, *p* < 0.001), with the covariates age (Wald *χ*^2^(5) = 67.54, *p* < 0.001), WHQ score (Wald *χ*^2^(5) = 20.29, *p* = 0.001), and the interaction between the covariates age and WHQ (Wald *χ*^2^(5) = 72.77, *p* < 0.001) as significant predictors of the effect. An increase in time to completion with an increase in ID was displayed (ID1: *M* = 2.73, SE = 0.06; ID2: *M* = 2.99, SE = 0.54; ID3: *M* = 3.33, SE = 0.06; ID4: *M* = 3.71, SE = 0.06; ID5: *M* = 3.88, SE = 0.06, ID6: *M* = 4.17, SE = 0.07). Pairwise comparisons demonstrated differences between all levels of difficulty (*p* < 0.001).

A significant interaction between condition and difficulty emerged (Wald *χ*^2^(10) = 25.50, *p* = 0.004), with the covariate age (Wald *χ*^2^(10) = 31.84, *p* < 0.001) and the interaction between the covariates age and WHQ score (Wald *χ*^2^(10) = 32.07, *p* < 0.001) as significant predictors. For the most part, an increase in time with an increase in difficulty in all three conditions. Pairwise comparisons are reported in Table 2.

**Table 2:** Pairwise comparisons from the interaction between condition and difficulty, with HSCT completion time as the dependent measure.

		Amplitude						Width						Combined					
		ID1	ID2	ID3	ID4	ID5	ID6	ID1	ID2	ID3	ID4	ID5	ID6	ID1	ID2	ID3	ID4	ID5	ID6
Amplitude	ID1		1.00	0.004	<0.001	<0.001	<0.001	1.00	1.00	0.460	<0.001	<0.001	<0.001	1.00	0.129	0.001	<0.001	<0.001	<0.001
	ID2			0.031	<0.001	<0.001	<0.001	<0.001	<0.001	1.00	<0.001	<0.001	<0.001	1.00	1.00	0.009	<0.001	<0.001	<0.001
	ID3				0.015	<0.001	<0.001	<0.001	<0.001	0.689	0.351	<0.001	<0.001	<0.001	1.00	1.00	<0.001	<0.001	<0.001
	ID4					<0.001	<0.001	<0.001	<0.001	<0.001	1.00	1.00	1.00	<0.001	<0.001	1.00	1.00	<0.001	<0.001
	ID5						<0.001	<0.001	<0.001	<0.001	<0.001	1.00	1.00	<0.001	<0.001	1.00	1.00	0.380	0.003
	ID6							<0.001	<0.001	<0.001	<0.001	<0.001	<0.001	<0.001	<0.001	<0.001	1.00	1.00	0.136
Width	ID1								<0.001	<0.001	<0.001	<0.001	<0.001	<0.001	<0.001	<0.001	<0.001	<0.001	<0.001
	ID2									<0.001	<0.001	<0.001	<0.001	<0.001	<0.001	<0.001	<0.001	<0.001	<0.001
	ID3										<0.001	<0.001	<0.001	<0.001	1.00	0.437	<0.001	<0.001	<0.001
	ID4											<0.001	<0.001	<0.001	<0.001	1.00	<0.001	<0.001	<0.001
	ID5												<0.001	<0.001	<0.001	<0.001	1.00	0.039	<0.001
	ID6													<0.001	<0.001	<0.001	1.00	1.00	<0.001
Combined	ID1														<0.001	<0.001	<0.001	<0.001	<0.001
	ID2															0.001	<0.001	<0.001	<0.001
	ID3																<0.001	<0.001	<0.001
	ID4																	<0.001	<0.001
	ID5																		0.004
	ID6																		

A significant interaction between space and difficulty also emerged (Wald *χ*^2^(5) = 11.24, *p* = 0.047), with the covariate age (Wald *χ*^2^(5) = 52.17, *p* < 0.001) and the interaction between age and WHQ score (Wald *χ*^2^(5) = 48.98, *p* < 0.001) as significant predictors. Pairwise comparisons revealed no difference between ID1 and ID2 in ipsilateral space (*p* > 0.05). All IDs in contralateral space were different (*p* < 0.05). When comparing between spaces, no differences emerged at ID1 and ID5. Furthermore, ID5 in ipsilateral space did not differ from ID4 in contralateral space and ID6 in ipsilateral space did not differ from ID5 in contralateral space.

### Hand Selection Complexity Task Errors

**Figure 4:**
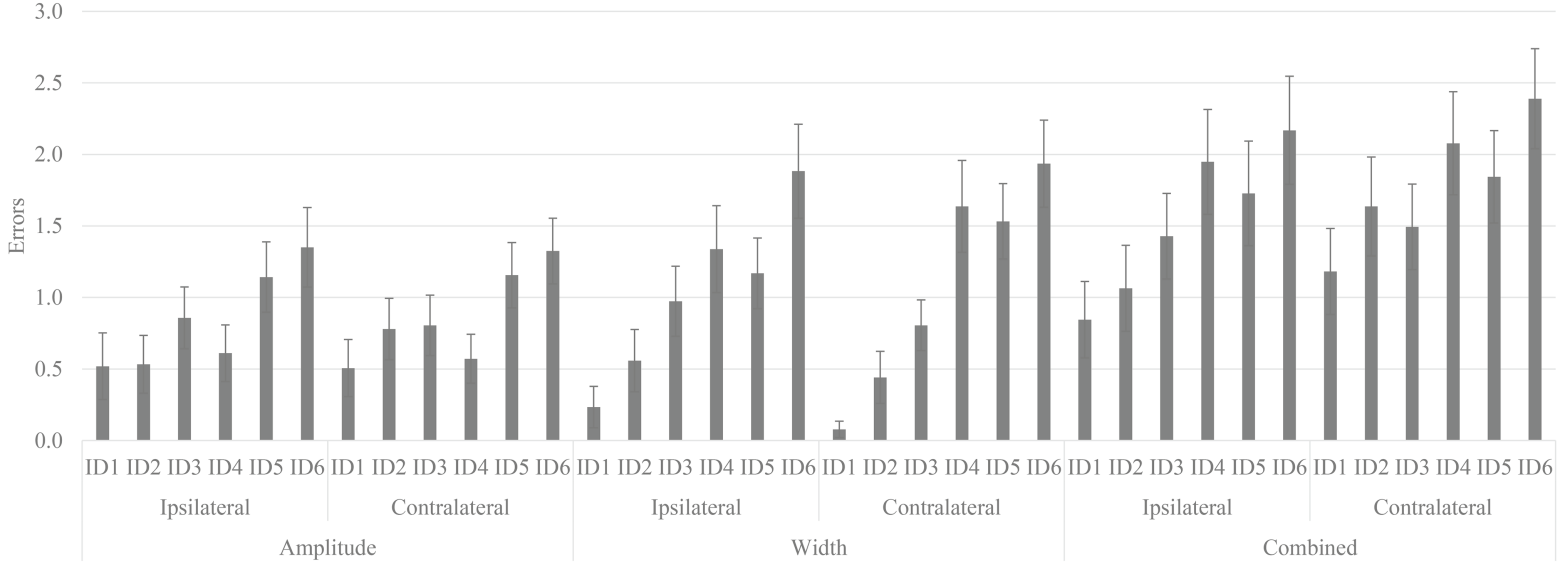
The three-way interaction between condition, space, and difficulty revealed distinct differences in the number of errors, which are discussed in the context of significant main effects and two-way interactions. Standard error bars are displayed.

An interaction between condition, space, and difficulty was revealed, when controlling for the covariate WHQ score (Wald *χ*^2^(10) = 18.76, *p* = 0.043; Figure 4). For clarity sake, only significant main effects and two-way interactions will be described in detail.

A main effect of condition (Wald *χ*^2^(2) = 13.84, *p* = 0.001) revealed a greater number of errors in the combined condition (*M* = 1.94, SE = 0.10) compared to amplitude (*M =* 1.17, SE = 0.08) and width (*M* = 1.35, SE = 0.08) conditions, which did not differ. Covariates age (Wald *χ*^2^(2) = 7.903, *p* = 0.019), WHQ score (Wald *χ*^2^(2) = 10.51, *p* = 0.005), and the interaction between age and WHQ score (Wald *χ*^2^(2) = 10.10, *p* = 0.006) were significant predictors.

The significant effect of difficulty (Wald *χ*^2^(5) = 34.87, *p* < 0.001) demonstrated an increase in error with increase in ID (ID1: *M* = 0.84, SE = 0.11; ID2: *M* = 1.16, SE = 0.13; ID3: *M* = 1.38, SE = 0.12; ID4: *M* = 1.69, SE = 0.14; ID5: *M* = 1.73, SE = 0.13; ID6: *M* = 2.13, SE = 0.14); however, the number of errors made in ID1, ID2, and ID3 did not differ. Covariates age (Wald *χ*^2^(1) = 10.41, *p* = 0.001), WHQ score (Wald *χ*^2^(1) = 4.65, *p* = 0.031), and the interaction between age and WHQ score (Wald *χ*^2^(1) = 6.03, *p* = 0.014) were significant predictors.

An interaction between condition and difficulty was found (Wald *χ*^2^(10) = 38.21, *p* < 0.001), with the covariates age (Wald *χ*^2^(10) = 20.58, *p* = 0.024), WHQ score (Wald *χ*^2^(10) = 36.88, *p* < 0.001), and interaction between age and WHQ score (Wald *χ*^2^(10) = 29.45, *p* = 0.001) as significant predictors. Pairwise comparisons are reported in Table 3.

**Table 3:** Pairwise comparisons from the interaction between condition and difficulty, with error as the dependent measure.

		Amplitude						Width						Combined					
		ID1	ID2	ID3	ID4	ID5	ID6	ID1	ID2	ID3	ID4	ID5	ID6	ID1	ID2	ID3	ID4	ID5	ID6
Amplitude	ID1		1.00	1.00	1.00	<0.001	<0.001	1.00	1.00	1.00	0.125	0.070	<0.001	1.00	0.235	0.046	<0.001	0.002	<0.001
	ID2			1.00	1.00	0.033	<0.001	<0.001	1.00	1.00	0.135	0.023	<0.001	1.00	0.147	0.012	<0.001	0.001	<0.001
	ID3				1.00	0.129	0.031	0.002	1.00	1.00	1.00	1.00	<0.001	1.00	1.00	1.00	0.001	0.096	<0.001
	ID4					0.002	0.009	0.398	1.00	1.00	0.429	0.224	<0.001	1.00	0.773	0.197	0.001	0.014	<0.001
	ID5						1.00	<0.001	0.126	1.00	1.00	1.00	0.850	1.00	1.00	1.00	1.00	1.00	0.143
	ID6							<0.001	<0.001	0.321	1.00	1.00	0.786	1.00	1.00	1.00	1.00	1.00	0.118
Width	ID1								1.00	0.001	<0.001	<0.001	<0.001	0.001	<0.001	<0.001	<0.001	<0.001	<0.001
	ID2									0.870	<0.001	<0.001	<0.001	0.149	0.001	<0.001	<0.001	<0.001	<0.001
	ID3										1.00	1.00	1.00	1.00	1.00	1.00	0.010	0.106	<0.001
	ID4											1.00	1.00	0.559	1.00	1.00	1.00	1.00	<0.001
	ID5												0.346	1.00	1.00	1.00	1.00	1.00	0.012
	ID6													<0.001	0.169	0.297	1.00	1.00	1.00
Combined	ID1														1.00	0.197	<0.001	0.004	<0.001
	ID2															1.00	0.018	1.00	<0.001
	ID3																0.815	1.00	0.006
	ID4																	1.00	1.00
	ID5																		0.193
	ID6																		

The interaction between space and difficulty was significant when controlling for the covariate WHQ score (Wald *χ*^2^(5) = 18.32, *p* = 0.003). Pairwise comparisons are reported in Table 4.

**Table 4:** Pairwise comparisons from the interaction between space and difficulty, with error as the dependent measure.

		Ipsilateral						Contralateral					
		ID1	ID2	ID3	ID4	ID5	ID6	ID1	ID2	ID3	ID4	ID5	ID6
Ipsilateral	ID1		1.00	<0.001	<0.001	<0.001	<0.001	1.00	0.362	<0.001	<0.001	<0.001	<0.001
	ID2			0.007	<0.001	<0.001	<0.001	1.00	1.00	0.004	<0.001	<0.001	<0.001
	ID3				1.00	0.301	<0.001	0.004	1.00	1.00	1.00	0.021	<0.001
	ID4					1.00	0.003	<0.001	1.00	1.00	1.00	0.906	<0.001
	ID5						0.061	<0.001	0.189	0.181	1.00	1.00	0.004
	ID6							<0.001	<0.001	<0.001	1.00	1.00	1.00
Contralateral	ID1								0.955	0.001	<0.001	<0.001	<0.001
	ID2									1.00	0.049	0.005	<0.001
	ID3										0.582	<0.001	<0.001
	ID4											1.00	0.023
	ID5												.055
	ID6												

### Hand Selection Complexity Task Switch Point

**Figure 5:**
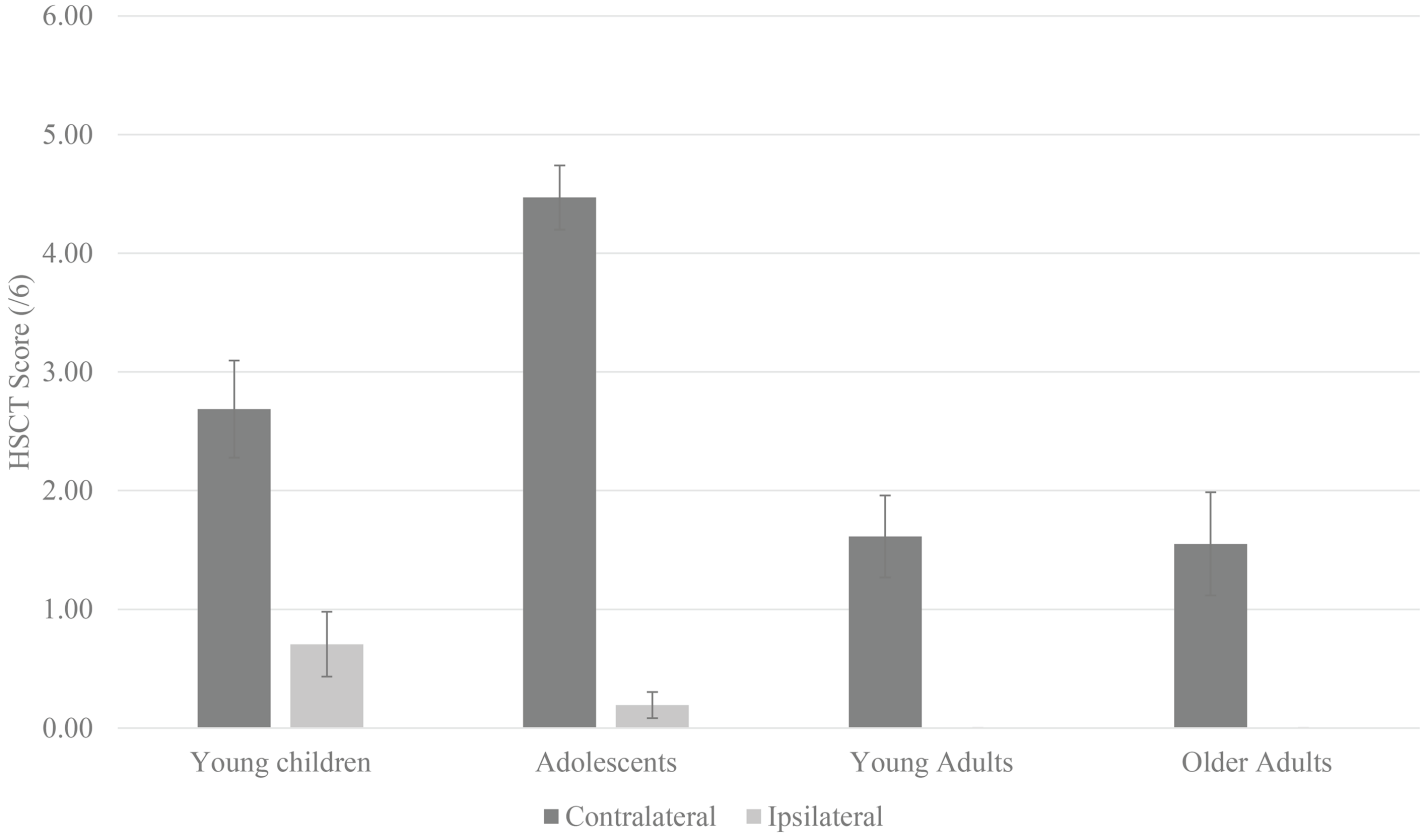
Age was a significant predictor of HSCT switch point. When controlling for WHQ score, the effect of space revealed a higher switch point in contralateral space.

The covariate age (Wald *χ*^2^(1) = 14.11, *p* < 0.001) was a significant predictor of HSCT switch point (see Figure 5). Furthermore, when controlling for WHQ score, the effect of space revealed a higher switch point in contralateral space (*M* = 2.92, SE = 0.20) compared to ipsilateral space (*M* = 0.23, SE = 0.08).

## Discussion

Taken together, the continuous covariate age was a significant predictor of main effects (space, difficulty) and interactions (condition × difficulty; condition × difficulty × space × WHQ) from analysis of HSCT completion time. Error data and hand switch data analysis revealed age as a significant predictor, overall. Furthermore, analysis of error data revealed the covariate age was a significant predictor of the effect of condition, and interaction between condition and difficulty. The covariate WHQ was also a significant predictor of HSCT completion time and error data, when considering the aforementioned effects and interactions.

As displayed in Figure 2, young children and older adults performed the HSCT slower than adolescents and young adults. Results suggest age is a significant factor in tasks that require fine motor control. Although it is to be expected that older children and young adults perform faster than young children in performance-based tasks (e.g., Kilshaw and Annett, 1983; Kail and Salthouse, 1994; Annett, 2002; Dellatolas et al., 2003; see Scharoun and Bryden, 2014, for review), findings in older adults are not concurrent with Gooderham and Bryden (2014), who revealed young adults and older adults performed similar in a related task. As discussed by Williams (2014), the results from the current work are more in accordance with Kalisch et al. (2006) who noted that, when performing tasks with fine motor control and dexterity requirements, older adults take longer than younger adults. The notion that fine motor movements see age-related declines in performance (slowing) with aging, especially in complex tasks, is by no means a novel concept (Seidler et al., 2010). Such observations are attributed to changes within central cognitive processes, which slow with increasing age. As a result, motor movements that require repetitive coordinated movements, like tapping, are generally affected (Kalisch et al., 2006; Seidler et al., 2010).

It is important to note that, older adults may have taken more time to perform the tasks; however, accuracy was not affected. Indeed, this aspect of performance was notably different from young children. Concurrent with Lamb et al. (2016), who had participants make alternating pen marks between a card located at the midline and a number of empty circles on a page, older adults were slower, but more accurate, compared to young adults. Related work with this age group has noted a tendency to sacrifice speed in order to ensure accuracy (Seidler et al., 2010). Older adults have thus been described as demonstrating a “play it safe” motor control strategy (Seidler et al., 2010). Because of this notion, it was expected that older adults would demonstrate more switches as the level of task difficulty increased; however, this was not the case.

Whereas the discussion of completion time and error data has focused around young children and older adults, switch point data of adolescents are of interest. Scharoun and Bryden (2014) reported that young children (ages 3–5) learn through exploring their environment, which hand is more skilled at particular tasks. As such, hand selection is associated with object proximity. With an increase in age (i.e., approximately ages 6–10), children identify which hand is most efficient; therefore, will select the preferred hand overwhelmingly. As evidenced in the present study, this pattern of hand selection is demonstrated even in cases where it may not be most effective to do so. Finally, between ages 10 and 12, a decreased reliance on the preferred hand, and subsequent increase in non-preferred hand performance, is interpreted as evidence of “adult-like” patterns of handedness beginning to emerge (Scharoun and Bryden, 2014).

Taken together, the Hand Selection Complexity Task was successful in identifying a significant effect of the covariate age on complexity switch-point. Taken in conjunction with results of HSCT completion time, and error data (i.e., interactions between condition, space, and difficulty level), it can be argued that task complexity does indeed play an important role when performing a task of increasing difficulty (Williams, 2014). At a certain point, task complexity will take precedent over object proximity and biomechanical efficiency in completing a movement with the preferred hand. This point is influenced by age-related effects, likely attributed to the development of handedness, and differences in strength of handedness. The decision to use the preferred hand in contralateral space, reflects the choice to cross the body’s midline, thus acting in contrast the biomechanical efficiency hypothesis (Gabbard et al., 1997). Findings thus add to existing knowledge of the role task complexity plays on hand selection (Williams, 2014). That said, the question of what task complexity really is, remains. In the current study, Fitts’ Law (Fitts, 1954) was used to quantify task complexity; however, as increasing the level of difficulty also reflects an increase in cognitive load (Liang et al., 2018), continued work in this area is needed to delineate what task complexity is and the best to way to measure it.

## Ethics Statement

This study was carried out in accordance with the recommendations of the Tri Council Policy Statement, Research Ethics Board at Wilfrid Laurier University with written informed consent from all subjects. All subjects gave written informed consent in accordance with the Declaration of Helsinki. The protocol was approved by the Research Ethics Board at Wilfrid Laurier University.

## Author Contributions

Data from this manuscript were included as part of NW’s master’s thesis; therefore, NW contributed to all aspects of this project, under the supervision of PB. SSB contributed to data analysis, interpretation, and preparing the manuscript for publication.

### Conflict of Interest Statement

The authors declare that the research was conducted in the absence of any commercial or financial relationships that could be construed as a potential conflict of interest.
